# Supplementary material for: Are carbon emissions trading and green financial instruments synergistic? -Comprehensive quantitative research based on content analysis
Source: PLoS One. 2024 Mar 7;19(3):e0298601. doi: 10.1371/journal.pone.0298601 (PMC10919601; doi:10.1371/journal.pone.0298601)
Supplement: S1 Data — (ZIP) [file pone.0298601.s001.zip › Data/地级市Z_F工作报告.zip!/╣π╬≈/╣≤╕█╩╨/└┤╫╘╣1⁄2╓┌║┼í╛┬φ┐╦╩2╛▌═°í┐_2016.docx]

各位代表：

现在，我代表市人民政府向大会报告工作，请予审议，并请列席会议的政协委员和其他同志提出意见。

一、过去五年工作回顾

五年来，在自治区党委、政府和市委的正确领导下，在市人大及其常委会的法律监督、工作监督和市政协的民主监督下，我们坚持以马克思列宁主义、毛泽东思想、邓小平理论、“三个代表”重要思想、科学发展观为指导，全面贯彻落实习近平总书记系列重要讲话精神，围绕建设西江流域核心港口城市目标，大力实施“工业兴市、工业强市”发展战略和“三年目标任务行动计划”“西江经济带项目建设三年攻坚计划”，深入开展“六个年”活动，经济社会保持平稳健康发展，较好完成了市四届人大一次会议确定的目标任务。

（一）经济实力持续提升

全市地区生产总值由2010年的545亿元提高到2015年的865.2亿元，年均增长7.4%；全社会固定资产投资由385.3亿元提高到778.6亿元，年均增长24%；财政收入由40亿元提高到72.7亿元，年均增长12.7%；城镇居民人均可支配收入由15531元提高到24890元，年均增长9.9%；农民人均纯收入由5289元提高到10017元，年均增长13.6%。

工业发展壮大。规模以上工业总产值由2010年的470.3亿元提高到2015年的855.9亿元，年均增长13.1%；规模以上工业增加值由168.7亿元提高到255.9亿元，年均增长8.6%；产值超亿元企业由101家发展到209家，建材、纺织服装与皮革、农林产品加工等产业产值突破100亿元，民用钢质船舶、服装、胶合板、水泥的产量在全区排名第1位。新能源汽车、电子信息、生物制药等一批新兴产业项目落户，建成了中国—东盟新能源电动车生产基地。产业园区基础设施不断完善，集聚力进一步增强，园区规模以上工业总产值由194亿元提高到483亿元，占全市规模以上工业总产值的56%。

农业稳步发展。粮食生产连年丰收，甘蔗、药材、木薯、水果等特色农业增产增效，建立“双高”糖料蔗基地3.7万亩，创建了现代特色农业（核心）示范区12个，“荷美覃塘”、亚计山生态养殖等示范区被评为广西现代特色农业（核心）示范区，“四季花田”示范区得到自治区领导的充分肯定。肉类总产量和生猪、家禽出栏量名列全区前茅，五个县市区均获全国生猪调出大县奖励。市级以上农业龙头企业由47家发展到121家，农民专业合作社由731家发展到1916家；家庭农场发展到1062家，排名全区第1位。建立富硒产品生产示范基地37个，获得自治区富硒农产品认定22个，富硒农业品牌成为全区标杆。农业耕种收综合机械化水平由33.4%提高到53.8%，水稻生产全程机械化走在全区前列。

服务业加快发展。社会消费品零售总额累计完成1600亿元，年均增长13.2%；建成大中型专业市场200多个、农贸市场300多个；发展电子商务企业150多家。2015年，港口货物吞吐量达5334万吨，稳居广西内河港口第1位。旅游接待总人数由627.1万人次增加到1444万人次，年均增长18.2%；旅游总收入由35.3亿元增加到137.6亿元，年均增长31.2%；3A级以上景区由2个发展到8个，桂平西山景区被评为“广西十佳旅游景区”。金融机构人民币存款余额973亿元，年均增长13.6%；贷款余额596.7亿元，年均增长16.4%。餐饮住宿、保险、快递等其他服务业稳步发展。

（二）城乡设施持续完善

开展城市建设大会战，累计投入420亿元用于城市建设，中心城区建成区由2010年的51.1平方公里增加到64.5平方公里，城镇化率由40.2%提高到46.5%。

城乡规划工作进一步加强。完成了城市总体规划局部修编、城区八大片区控制性详细规划编制和城市设计，完善了道路综合交通、绿地系统、排水等28个专项规划，完成了桂平、平南城区和39个乡镇总体规划以及1379个村屯规划。

城市建设管理成效明显。新建了迎宾大道北延段、建设路西延段等6条城市道路，完成了中山路、金港大道等5条主干道综合改造，打通了桂林路、仙衣路等城区“断头路”，改造小街小巷180多条，青云大桥、贵港城区至覃塘一级路、解放路和同济大道下穿铁路工程、贵港高铁站前广场等项目扎实推进，罗泊湾大桥、东环路即将建成。完成了马草江公园配套工程和东湖公园改造工程，民族文化公园建成向市民开放。加强城市绿化亮化彩化，提升改造绿地104.4公顷。“智慧城市”建设取得初步成果，“天网工程”竣工投入使用，市中心城区主要公共场所开通免费WiFi。开展市容市貌整治和打击“双违”活动，中心城区、桂平城区、平南县城面貌焕然一新。

交通设施逐步完善。南广高铁和梧州至贵港、桂平至来宾等高速公路通车，新增等级公路1159公里，四级以上公路总里程突破7000公里，实现了乡乡公路联网、行政村基本通水泥（或沥青）路。加快港口现代化建设，实施西江经济带基础设施项目建设大会战，大藤峡水利枢纽、贵港航运枢纽二线船闸等工程开工，郁水作业区、武林港作业区等项目竣工，实现了县县通高速铁路、高速公路和高等级航道。

农村生产生活条件得到改善。投入39.4亿元用于水利工程建设，新增达标防洪堤21公里，完成病险水库除险加固109座、中小河流整治61公里，改造或硬化灌区渠道1518公里，新增、恢复或改善灌溉面积147万亩。投入5.3亿元建设桂平市和覃塘区整县推进高标准基本农田项目39个。建成农村饮水工程1089项，解决了270万农村人口饮水安全问题。投入9.1亿元用于水库移民安置区基础设施建设和经济发展，受益移民19.4万人。投入36.1亿元用于城乡电网建设，19座110千伏及以上变电站建成运营。

（三）改革开放持续深化

重点领域改革加快推进。强化行政审批串联转并联、前置转后审、零散转集中等“三个转变”，取消、下放、调整行政审批项目794项，在国土、住建、环保部门试点“说不”报告制度。全面落实“先证后照”，推行“三证合一”“一照一码”，启动“全城通，就近办”，累计发展市场主体10.8万户。事业单位分类改革、综合行政执法改革、乡镇“四所合一”改革、不动产统一登记改革稳步推进。财政管理体制改革成效明显，盘活财政存量资金26.7亿元；落实营改增、促进小微企业发展等各项税收优惠政策，减免税收22.5亿元。加快国有企业改革，国有资本及收益连续5年增长。供销合作社综合改革走在全区前列，荣获全国“金扁担奖”。农村土地经营权确权登记工作有序开展，卫生、水利、林权、园区、公务用车等改革取得新成效。

招商引资取得新突破。实行全民招商、重力招商，深入开展“大招商、招大商”活动，累计引进项目1324个，合同投资额1862.9亿元，到位资金1157.2亿元；西江重工基地、理昂生物质发电、益海嘉里粮油加工、保利高塑胶制品等一批大项目落户贵港，引进了华奥、赛尔康等一批重点企业。

开放合作取得新成效。外贸进出口总额累计85亿元，年均增长19.5%，胶合板出口占广西的70%以上；累计利用外资2.9亿美元，年均增长20.2%。区域合作进一步加强，与广东广业公司合作建设的粤桂（贵港）热电循环经济产业园，被列为两省区重点推进项目；与玉林市签订了《玉贵区域一体化发展战略合作框架协议》。对外友好交流进一步密切，与韩国首尔特别市九老区、菲律宾三宝颜市建立了友好城市关系。

（四）生态环境持续改善

污染物减排成效明显，2015年化学需氧量、氨氮、二氧化硫和氮氧化物排放量比2010年分别下降16.5%、15%、11.7%、34.8%，全面完成自治区下达的主要污染物减排目标任务。完成规模化畜禽养殖污染减排项目592个。强化大气污染防治，城市空气质量总体良好。节能降耗成效突出，2015年单位地区生产总值能耗比2010年下降19.7%，规模以上万元工业增加值能耗下降32.3%。水资源保护扎实推进，主要江河断面水质均达国家Ⅲ类水质标准，城市集中式饮用水源地水质达标率98.4%。深入开展“美丽贵港”活动，清洁乡村、生态乡村扎实推进。实施“绿满八桂”造林绿化工程，累计完成山上造林84万亩，建成自治区级示范绿化村屯667个，森林覆盖率达46.3%。建成农村户用沼气池2.1万座。市生活垃圾焚烧发电厂投入运营，建成乡镇垃圾中转站58座，生活垃圾无害化处理率达100%。建成农村污水处理项目71个，农村环境连片整治示范项目89个，受益人口42.5万人。

（五）民生保障持续增强

累计投入民生资金569亿元，占财政支出的80.5%。千方百计促进就业，累计新增城镇就业8.6万人，新增农村劳动力转移就业21.4万人，城镇登记失业率控制在4.3%以内。建立了覆盖城乡居民的社会保障体系，社会保险参保总人数达288.8万人次，比2010年翻了一番；企业退休人员月人均养老金从2010年的1070元提高到2015年的1824元，城乡居民养老保险基础养老金标准从每人每月55元提高到90元。建立被征地农民养老保险和最低生活保障制度。投入51.6亿元，建设保障性住房4.8万套，完成农村危房改造5.5万户，帮助20多万住房困难群众圆了安居梦。累计投入救助资金25.3亿元，救助城乡低保、五保供养等对象309.7万人次。投入各类救灾资金9000多万元，为各类优抚对象发放抚恤金、定补金3.6亿元。发放教育各类资助金12.6亿元，资助学生176万人次。建成乡镇敬老院76个、五保村559个、农村幸福院255个，为80周岁以上老人发放了高龄津贴。脱贫攻坚扎实推进，投入14.2亿元用于脱贫攻坚，修建贫困村屯道路1229公里，扶持发展经济作物40万亩，扶持养殖畜禽162万头（羽），28.2万贫困人口实现脱贫。

（六）社会事业持续进步

教育事业加快发展。投入26.3亿元用于学校建设，新建荷城初中、港南二中、龚州中学、桂平五中、金港小学、港宁小学等学校，改扩建18所高中，68所乡镇公办中心幼儿园投入使用。学前三年毛入园率达77%，义务教育巩固率达94.8%，每万人口高中在校生数排全区第1位，提前一年实现自治区示范性普通高中县域全覆盖。职业教育加快发展，成功引入广西工业职业技术学院办学，启动西江职教园区建设。民族教育、特殊教育、民办教育等各类教育稳步发展。

科技创新能力得到增强。获市级以上科技成果奖励150项，建成市级以上科技创新平台101个，累计获得国家授权专利1554件；科技进步贡献率达43.5%，比2010年提高了5.1个百分点。

文化体育事业繁荣发展。市文化艺术中心开工，市图书馆、博物馆、档案馆建成投入使用，建成村级公共服务中心452个、农家书屋1135个，公共文化基础设施场所全部免费开放；村村通广播电视综合覆盖率达98.2%；群众文化活动深入开展，“荷城”城市品牌影响力不断增强。加强社会主义精神文明建设，“和为贵、诚为本、干为先”的贵港精神深入人心。荣获第六轮“自治区文明城市”，并获“全国文明城市提名城市”。全民健身运动深入开展，市体育中心建成投入使用。

卫生计生工作得到加强。投入11.9亿元建设228个卫生基础设施项目，市人民医院外科楼、精神卫生医院、全科医生培养基地建成投入使用，新农合平均参合率达98%。诚信计生扎实推进，人口自然增长率控制在9‰以内。

其他事业统筹发展。实施“质量强市”成效明显，获中国驰名商标2个、地理标志产品10个，广西著名商标62个、名牌产品34个。加强应急管理工作，公共安全应急和救援能力进一步提升。食品药品监管得到加强。“平安贵港”建设扎实推进，社会和谐稳定，人民群众安全感和满意度跃居全区前列。全面完成“六五”普法教育，矛盾纠纷调解和信访工作成效明显。强化安全生产工作，安全生产形势总体保持稳定。加强“双拥”工作，实现“全国双拥模范城”八连冠和“自治区双拥模范城”九连冠。妇女儿童、老年人和残疾人权益得到有效保障，民族、宗教、侨务和对台工作取得新成效，档案、地方志、社会科学等事业取得新成绩，防灾减灾、气象服务、人防建设得到加强，统计、绩效、物价、公积金、机关后勤、二轻联社等工作取得新进步，工会、共青团、妇联、工商联、科协等组织在促进经济社会发展中发挥了积极作用，海关、海事、检验检疫、边检、武警、消防、农垦、邮政、通信、水文、石油化工、烟草、盐业、船舶检验、地质勘探、无线电监测等中区直部门为经济社会发展提供了强有力支持。

（七）政府建设持续加强

依法行政扎实推进。加强法治政府建设，规范行政权力运行，公开部门权力清单，严格规范行政执法行为，全面实施政府法律顾问制度。坚持科学决策、民主决策、依法决策，健全公众参与、专家论证、风险评估、合法性审查和集体讨论决定等政府决策程序。

党风廉政建设得到加强。严格贯彻执行中央“八项规定”精神和国务院“约法三章”，深入开展党的群众路线教育实践活动、“三严三实”专题教育和“两学一做”学习教育，加强行政监察和审计监督，廉政建设和反腐败斗争取得新成效，查处了一批发生在群众身边的“四风”和腐败问题。

政务服务进一步强化。加快电子政务建设，深入推进政务公开和政府信息公开，不断提高政府工作透明度。推进政务服务提速增效，实现市、县、乡、村四级政务服务体系全覆盖，政务服务水平进入全区先进行列。畅通政民互动渠道，建立完善市长热线电话及微信、市政微信、政务微信、网络问政平台，积极回应群众关心的热点问题。持续开展“市长服务企业接待日” 活动，千方百计为企业排忧解难。

认真接受监督。坚决执行市人大及其常委会决议和决定，自觉接受市人大及其常委会的法律监督、工作监督和市政协的民主监督，以及社会和舆论监督。坚持重大事项向市人大常委会报告、向市政协通报，人大代表建议和政协提案办结率100%。

各位代表！过去五年的成绩来之不易。这是在自治区党委、政府和市委的正确领导下，在市人大及其常委会的法律监督、工作监督和市政协的民主监督下，在市中级人民法院和市人民检察院的司法保障下，全市各级各部门和548万各族人民团结奋斗的结果，历届党委、人大、政府、政协为我们打下了良好基础，社会各界朋友给予了大力支持帮助。在此，我谨代表市人民政府，向全市各族人民，向人大代表、政协委员，向人民法院和人民检察院，向各民主党派、工商联、无党派人士、各人民团体，向中区直单位和驻贵人民解放军、武警部队官兵，向所有关心支持贵港发展的社会各界人士，致以崇高的敬意和衷心的感谢！

五年实践证明，推动贵港经济社会持续健康发展，必须坚持党的领导，坚决贯彻执行党的路线、方针、政策，切实把自治区党委、政府和市委的各项决策部署落到实处；必须走改革创新之路，勇于探索，敢为人先，着力解决发展中深层次的矛盾和问题；必须坚持发展第一要务，全力以赴抓工业，不断优化产业结构，提高经济质量效益；必须突出抓好招商引资，深化开放与合作，不断增强发展的后劲和动力；必须坚持把保障和改善民生放在首位，深入推进城乡统筹发展，让改革发展成果惠及广大群众，增强人民群众获得感；必须加强政府自身建设，强化工作落实，提高政府执行力，努力创造良好的发展环境。这些经验是指导我们今后做好各项工作的宝贵财富。

我们也清醒地认识到，受宏观环境、发展阶段、经济结构等多重因素影响，我市发展存在的问题不容忽视。主要是综合经济实力还不够强，总量不大，财力薄弱，全面建成小康社会任务艰巨；经济增长方式还比较粗放，经济结构有待优化，工业短板突出，传统增长动力减弱，新的增长动力不足，农业现代化和城镇化水平不高；开放合作广度和深度不够，招商引资工作仍需进一步加强；基本公共服务发展滞后，社会保障体系仍不完善，脱贫攻坚任务仍比较重；节能减排压力大，生态环境改善更加紧迫。此外，政府自身建设仍需加强，反腐倡廉任重道远，干部作风尚需进一步转变。我们要不断改进和提升政府工作水平，用新理念、新办法不断解决前进中的各种矛盾和问题。

二、今后五年工作

今后五年，是我们实施“十三五”规划、决胜小康、再创辉煌的关键期，也是加快发展、赶超跨越的战略机遇期。根据中国共产党贵港市第五次代表大会精神，今后五年政府工作的总体要求是：高举中国特色社会主义伟大旗帜，全面贯彻党的十八大和十八届三中、四中、五中全会精神，以马克思列宁主义、毛泽东思想、邓小平理论、“三个代表”重要思想、科学发展观为指导，认真贯彻习近平总书记系列重要讲话精神，深入推进“四个全面”战略布局，牢固树立创新、协调、绿色、开放、共享的发展理念，全面落实自治区“四大战略”“三大攻坚战”的决策部署，按照市委“13446”工作思路，全力推进实力贵港、活力贵港、魅力贵港、给力贵港建设，大力实施“工业兴市、工业强市，改革引领、创新驱动，东进融合、开放带动”三大战略，坚决打赢“贵港经济翻身仗、城市提升主动仗、脱贫摘帽攻坚仗、作风建设持久仗”四场硬仗，围绕“工业大发展、项目大兑现、服务大提速、城市大变化、县域经济大突破、脱贫攻坚大成就”六大目标，积极推进港产城互动融合发展，加快建成西江流域核心港口和新兴工业城市，全面建成小康社会，实现赶超跨越，再创贵港新辉煌。

今后五年，我市经济社会发展的奋斗目标是：到2020年，地区生产总值突破1400亿元，年均增长9%以上，比2010年翻一番以上；三次产业结构调整为12：45：43；财政收入突破134亿元，年均增长13%以上；固定资产投资突破1600亿元，年均增长18%以上；规模以上工业增加值突破520亿元，年均增长12%以上；社会消费品零售总额突破600亿元，年均增长9%以上；港口货物吞吐量突破8000万吨，年均增长8.5%以上；外贸进出口总额突破33亿元，年均增长10%以上；城乡居民人均可支配收入突破24800元，年均增长7.8%以上，比2010年翻一番以上。脱贫攻坚战取得全面胜利，到2018年，29.6万农村贫困人口实现脱贫。

为实现上述目标，我们将重点做好以下几方面：

（一）加快产业发展，着力提升经济实力

壮大工业经济。围绕建设西江流域新兴工业基地，实施“工业兴市、工业强市”战略，力争到2020年，规模以上工业总产值1800亿元以上。

发展战略性新兴产业。认真落实《中国制造2025》行动计划，培育发展新能源汽车和电动车、电子信息、现代船舶、航空产业零部件、智能专用设备制造、生物制药、节能环保、新材料、新能源等战略性新兴产业。抓好华奥新能源汽车产业园、赛尔康电子信息产业园等项目建设，打造广西第二个汽车（新能源）生产基地，壮大中国—东盟新能源电动车生产基地。到2020年，新兴产业产值突破1000亿元，形成区域竞争力较强的战略性新兴产业集聚地。

推动产业提档升级。提升自主创新能力，支持现有企业技术改造和设备更新，推动传统产业优化升级和高效发展。完善和落实扶持企业发展政策，设立产业发展基金、园区发展基金，破解企业发展瓶颈制约，稳定现有企业生产经营。实施“工业扶优扶强”和“抓大壮小扶微”工程，培育壮大骨干企业，到2020年，全市规模以上企业突破800家，建材、纺织服装与羽绒、农产品食品加工、造纸与竹木材加工产业产值分别突破200亿元，生物化工、冶金、电力等产业产值分别突破100亿元。

建设现代化园区。加强园区规划，扩大园区规模，优化园区空间和产业布局，不断提升园区承载力，增强产业洼地吸聚效应，推动产业向园区集聚。加强土地储备，依法清理闲置土地。加快园区水、电、路、污水处理等基础设施和公租房、医院、商业、休闲等配套设施建设，完善信息、技术、创业、培训、融资、展览展示等公共服务功能。在市产业园区和桂平、平南工业园区建设产业转移转型升级示范区，把西江产业园建成“双创”生态科技示范园，支持县市区扩园修编。到2020年，园区工业总产值突破1400亿元，占全市工业总产值比重70%以上。

推进农业现代化。加快转变农业发展方式，提升农业质量效益，建设西江流域现代农业强市。五年内，农林牧渔业增加值年均增长4%。毫不放松粮食生产，推进高标准基本农田建设，增强粮食生产能力，确保粮食年总产量保持150万吨以上。扩大特色农业规模。全面推动区、市、县、乡四级现代特色农业示范区创建工作，重点推进港北区国家级现代农业示范区和农业综合开发产业园区建设，到2020年，每个乡镇建成现代特色农业示范区1个以上。进一步发展肉蛋鱼禽、果菜菌茶等优势农产品，打造珠三角地区绿色农产品生产基地和配送中心。稳定糖料蔗种植面积，建成“双高”糖料蔗示范基地5万亩。做大农业品牌，建立20万亩富硒优质稻产业基地和一批富硒蔬菜、水果、茶叶、畜禽等示范基地，建设广西乃至全国具有影响力的“中国硒港”，打响富硒农业品牌。健全现代农业经营体系，到2020年，市级以上农业龙头企业突破180家、农民专业合作社突破3000 家、家庭农场突破1500家。发展“订单农业”，培育多元化市场流通主体，搭建农超、农商、电商对接平台，拓宽农产品销售渠道。发展科技农业，以建设智慧农业为抓手，强化现代农业科技创新和运用，创建一批生态农业科技园、示范区、示范带。推进农业生产机械化，到2020年，农业耕种收综合机械化水平73%以上。完善水利设施，推进大藤峡灌区工程建设，完成5座病险水库除险加固、15座沿江防洪病险水闸加固和20处中小河流治理，新增或改善灌溉面积20万亩以上。

发展现代服务业。把现代服务业作为经济转型升级的战略取向，构建具有贵港特色和持续竞争力、支撑力、带动力的现代服务业产业体系。

打造西江流域商贸物流中心。依托贵港作为国家二级物流园区布局城市优势，规划建设西江沿江港口物流带。推动开通贵港港口至北部湾港口的江铁海联运新通道，建成区域性航运枢纽、江铁海物流集疏中心，把贵港港打造成为北部湾港口的唯一后方供给港。推进苏湾物流园、罗泊湾物流园、江南物流园等建设。建成红星美凯龙家居建材市场、上亿国际汽贸博览城、林产品交易产业园、农产品物流中心等大型专业市场。继续培育贵港至香港定期航班，发展集装箱运输。积极推进西江航运交易所建设，搭建港口货物交易信息平台。

打造西江流域旅游目的地。建设一批特色旅游名县名镇名村，推进桂平市创建广西特色旅游名县，打造平南县全域旅游示范区、港北区健康养老生态旅游农业示范区、港南区生态旅游示范区、覃塘区休闲养生旅游农业示范区。加强与珠江—西江经济带各城市的旅游合作，推动沿路、沿江旅游带建设。打造重点景区景点，加快太平天国金田起义遗址、北帝山生态旅游区、平天山国家森林公园旅游综合区、铜鼓湾温泉生态度假村、九凌湖旅游度假区等重大旅游项目建设。积极挖掘和开发具有地方特色的旅游商品，培育一批旅游商品企业。五年内，旅游接待人数年均增长18%以上，旅游总消费年均增长25%以上。

培育新兴服务业。大力推进“荷城电商”计划，实施电子商务进农村综合示范工程，创建一批国家级、自治区级电子商务示范镇、示范村，重点打造桂平市国家级电子商务进农村示范县。优先发展信息、金融、保险、中介服务等生产性服务业，加快发展家政、养老、社区服务等生活性服务业，提高第三产业在国民经济中的比重。

做大县域经济。各县市区立足资源优势，明确产业重点，打造产业集群，彰显区域特色。桂平市重点发展节能环保新型陶瓷、生物化工、农副产品加工、纺织服装、旅游等产业。平南县重点发展建材、造纸与林板加工、生物科技、碳酸钙新材料等产业。港北区重点发展热电循环、电子信息、电动车、生物制药、石墨烯新型材料、现代服务业和现代城郊农业。港南区重点发展林产品、羽绒深加工、现代特色农业和现代物流等产业。覃塘区重点发展新能源汽车、新能源船舶制造、装备制造等战略性新兴产业。到2020年，县域经济总量排位大幅提升，桂平市进入全区前5名，平南县进入前10名，港北区、港南区和覃塘区比2015年进步20名。

推动科技创新。实施创新驱动发展战略，增强科技对经济发展的支撑能力，到2020年，科技进步贡献率达50%以上。加大研发支持力度，设立科技创新发展基金，实施发明专利倍增计划和科技成果转化大行动，实现每万人口发明专利拥有量1件以上，重大科技成果转化应用并产生明显经济社会效益50件以上。建设科技创新平台，建成国家级高新技术产业开发区和国家级科技企业孵化器。开展引智引技活动，鼓励支持企业引进新技术、开发新产品。积极培育与重点产业发展相配套的公共服务平台，建设新能源汽车产业技术研究院和低速电动车产业发展研究中心。积极开展以“科技进企业”“科技进社区”“科技下乡”为重点的科普活动。实施“质量强市”战略，推动企业质量品牌建设和产品标准化生产。

（二）深化改革开放，全面增强发展活力

推进重点领域改革。抓好财税改革，实行中期财政规划管理，加大一般公共预算、政府性基金预算、国有资本经营预算的统筹力度，完善政府债务管理，防范财政风险。加快公立医院综合改革，降低群众医疗费用。深化园区管理体制改革，发挥园区作为工业发展主战场作用。全面启动新一轮国资国企改革工作，确保国有资产保值增值。加大土地确权登记颁证工作，深化水权水价水市场改革。继续分类推进事业单位改革，统筹抓好金融、投融资、教育、养老、司法、住房、供销社、公共资源交易、集体林权、国有林场的改革。

强化招商引资。实行全民招商、重力招商不动摇，继续开展“大招商、招大商”活动，招商引资实际利用资金总量突破2000亿元，年均增长12%以上。突出招大引强，瞄准世界500强、国内百强企业和知名央企、民企开展招商，到2020年，引进投资额超亿元项目500个以上。突出产业链招商，围绕新能源汽车、电动车、电子信息、生物医药等产业，着力引进投资额大、技术含量高、产业关联度强的新兴产业。突出重点区域招商，积极承接港澳台、珠三角、长三角、京津冀等发达地区产业转移，引进先进产能和技术。突出项目落实，完善项目服务机制，强化要素保障，破解项目建设瓶颈制约，提高项目履约率。

加强区域合作。围绕建设西江经济带对外开放的重要节点城市、物流枢纽城市、粤桂黔高铁经济带产业转移前沿基地，突出向东开放，主动融入“一带一路”、中国—东盟合作、泛珠三角区域合作，加强与广州、深圳、佛山、东莞等重点市（区）合作。强化与梧州、玉林的合作，推进梧玉贵一体化发展。加快建设粤桂（贵港）热电循环经济产业园，打造两省区合作典范。

扩大对外贸易和交流。实施加工贸易倍增计划，积极引进境外项目、技术、资金，鼓励支持外贸企业实施品牌战略，整合优势产业和技术走出国门。加快西江保税物流中心（B型）、电子口岸和广西国际贸易“单一窗口”建设。加强港口物流合作，扩大与东盟的贸易往来，开拓东盟新能源汽车国际市场。密切对外交流合作，争取与一批规模匹配、地位相当、优势互补的外国城市建立国际友好城市关系。

（三）着力夯实基础，加快推进城乡建设

加快城市建设。搭建城市发展大框架，谋划城市发展新格局。

拉大城市框架。坚持规划先行，高标准修编城市总体规划、控制性详细规划和专项规划，完善城市空间发展布局。围绕构建西江流域核心港口和新兴工业城市发展全新格局，着力推进中心城区“一江两岸、一个功能主体、三大功能板块、六大城市组团”空间功能性布局和规划建设，规划启动城市新区建设。开工建设江南大桥、城东大桥、岭蒙大桥，加快港区大道、江南大道、城北大道、郁林大道等城市道路建设。将覃塘区纳入中心城区总体规划，加快发展。到2020年，中心城区建成区面积达到80平方公里，人口规模达到80万人。

打造宜居城市。深入开展“美丽广西·宜居城市”建设活动，完善城市功能，打造靓丽之城，到2020年城市宜居指数80%以上。实施城市道路“白改黑”改造，打通城区“断头路”。加快海绵城市建设，城市建成区20%以上的面积达到将70%的降雨就地消纳和利用。加快地下综合管廊建设，力争建成地下管廊20公里。加快城市绿化亮化和美化。推进郁江两岸滨水地带景观改造和堤路园综合治理工程建设，打造临江城市景观；建设园博园、鲤鱼江湿地公园、北山公园、南湖公园等一批公园，城区绿化覆盖率达36%以上；亮化重点区域，消除中心城区“无灯”区。改扩建供电供气管网，规划建设中心城区第二水源，中心城区供水普及率95%以上。积极发展城市公交事业，城市公交分担率20%以上。改造旧城区基础设施、棚户区、城中村和农贸市场，让老城区焕发新活力。

强化城市管理。理顺城市管理体制，推动城市管理向城市治理转变。加快智慧城市建设，整合市政公用、交通、公共服务、能源、人口、商务、政务、消防等城市管理信息资源，构建统一的城市公共基础数据库和公共信息服务平台。开展市容市貌整治，解决 “城市病”问题。严厉打击“双违”，进一步规范城乡建设秩序。

抓好城镇和农村建设。推进县城建设。加快桂平、平南城区建设，不断提升县城承载力，到2020年，桂平市城区面积达45平方公里、人口50万人；平南县城区面积达35平方公里、人口35万人。加强城镇建设，到2020年，城镇化率达55%以上。实施百镇建设示范和少数民族乡示范项目，扶持沿江、沿路交通枢纽特色小镇发展。创新城镇新区开发模式，有序推进农村人口向城镇流动和农业转移人口市民化。坚持产业兴镇，打造桥圩羽绒、庆丰富硒、木乐服装、蒙圩陶瓷、平山竹芒、覃塘莲藕等产业，争取一批特色镇列入自治区级产城互动发展试点。持续开展“美丽贵港”活动，巩固“清洁乡村”成果，推进“生态乡村”“宜居乡村”“幸福乡村”建设。

完善交通网络。构建全方位、多功能的江铁海、水陆空交通联运体系，形成以贵港为核心的“一小时高效联运圈”。加快高速公路建设，建成贵港至隆安、贵港至合浦、柳州至梧州、荔浦至玉林高速公路（贵港段），形成“三横二纵一环”高速公路网；规划建设贵港西外环等高速公路，加快高速公路与城市主干道的衔接，构建“二环七射”高等级城市快捷路网。改造市中心城区西环路。改造省道干线，实施贵港至黎塘、贵港至梧州、贵港至玉林（贵港段）一级公路扩建工程，建成金田至蒙山、社步至兴业、四灵至大岭等二级公路，到2020年通二级以上公路乡镇达85%以上。加快农村公路提级改造，实现乡乡通三级以上公路、村村通四级以上公路。提高港口集疏运能力，推进大藤峡航运枢纽一线船闸、贵港航运枢纽二线船闸等工程建设，开工建设贵港至梧州一级航道、桂平至来宾二级航道、贵港至北海城际铁路和柳州至肇庆普通铁路（贵港段），打通与北部湾海港联运通道，实现江铁海联运。顺应现代服务业发展需要，规划建设航空港。

推进生态建设。树立绿色发展理念，落实环境保护“党政同责，一岗双责”，严格执行《环境保护法》，深入贯彻落实国务院“大气十条”“水十条”“土壤十条”，严守生态红线，严格环境准入，确保实现环境质量改善的总体目标。倒逼产业结构转型升级，严格落实能源消费总量和“双管控”措施，发展循环经济。积极推广使用清洁能源。加强大气污染治理，确保大气环境质量优良。实施直排污水截流工程，城区生活污水处理率达95%以上。加快乡镇污水垃圾处理设施建设，实现镇镇有污水处理厂和垃圾转运站。推进农村环境连片整治，开展水土保持生态建设。推进水系连通及城市生态补水工程，构建沿江绿色生态廊道及生态水网。加快国家森林城市、国家园林城市建设，到2020年，森林覆盖率达46.5%以上。

（四）坚持精准施策，坚决打赢脱贫攻坚战

千方百计促进贫困农民增收。实施“特色产业富民行动”，推进“1+N”产业扶贫，鼓励和引导贫困村群众因地制宜发展特色产业，到2020年，每个县市区发展2-5个特色优势产业，每个贫困村形成1个以上主导产业。实施“千企带万家脱贫行动”，强化龙头企业、农民专业合作社与贫困农户利益联结，推广“公司+合作社+农户”模式，确保每个贫困村、每个贫困户至少有一家企业或合作社带动。实施“劳动就业转移脱贫行动”，加大贫困群众职业技能培训力度，提高贫困家庭劳动力转移就业和自主创业能力，引导贫困群众进城务工、转移就业。推进资产收益扶贫，推广土地、房屋、财政扶贫资金和扶贫小额信贷资金入股合作等经营模式，拓宽贫困群众增收渠道。

加快推进贫困地区设施建设。加大财政专项扶贫资金投入，建立扶贫资金项目整合管理平台、扶贫融资平台，鼓励引导金融资金和社会资金投入扶贫开发。建立以精准扶贫为导向的资金使用管理机制，各级财政按照当年一般公共预算收入增量要求的比例安排专项扶贫预算，按当年规定清理收回的存量资金中可统筹使用资金的50%以上用于扶贫开发。实施道路硬化工程，抓好尚未通硬化道路的行政村和20户以上自然屯通屯道路建设，解决贫困群众出行难、运输难问题。实施饮水巩固提升工程，解决农村贫困群众饮水安全问题。实施农网升级改造工程，解决贫困村屯、贫困户生产生活用电问题。实施信息网络联通工程，实现自然屯宽带信息网络全覆盖。实施易地扶贫搬迁工程，五年内完成易地搬迁贫困人口1.7万人。

全面提升贫困群众保障水平。推进教育精准扶贫，统筹教育资金和项目向贫困地区倾斜。免除贫困家庭高中阶段学生学杂费，实行12年免费教育。落实职业教育资助政策，让未升入普通高中的贫困家庭学生都能进入中等职业学校就读。深入实施健康扶贫工程，推进贫困地区县乡村三级医疗卫生服务网络标准化建设。加大医疗救助力度，逐步提高贫困人口的医保补助、门诊和住院治疗费用报销标准，将贫困人口全部纳入重特大疾病救助范围。推进低保制度与扶贫开发政策有效衔接，充分发挥社会救助在脱贫攻坚中的政策性保障兜底作用，2017-2020年农村最低生活保障标准年均增长15%左右。

强化精准脱贫责任。落实“一把手”脱贫攻坚责任制，完善考核、评估、退出等机制。建立“双认定”台账，实行挂图作战、清单管理、滚动集成、精准摘帽的管理模式。发挥扶贫大数据平台作用，推动市、县、乡、村四级扶贫数据信息交换共享。加强扶贫队伍建设，成立乡镇、村屯扶贫工作站，充实扶贫专干，确保专人落实脱贫攻坚工作。建设社会扶贫对接平台，鼓励各类企业、社会组织、个人参与脱贫攻坚，形成政府、市场、社会协同推进的大扶贫格局。严格执行通报、督查和协调三项制度，确保脱贫摘帽各项措施落到实处。

（五）强化民生保障，统筹发展社会事业

加快教育事业发展。实施学前教育第二、三期行动计划，学前三年毛入园率达90%。加快义务教育学校标准化建设，实施薄弱学校改造计划和中小学校舍维修等项目，建设荷城二中、荷城三中、荷城六小等城区学校，推进桂平、平南城区学校建设，基本消除义务教育学校“大班额”和寄宿学校“大通铺”。县市区全部通过义务教育发展基本均衡县评估，义务教育巩固率达96%。启动贵港高中新校区建设，新建高中2所，改扩建高中16所，高中阶段教育毛入学率达90%。加快西江职教园区建设，引进高等职业院校1-2所，引导中等职业学校入园发展，打造具有贵港特色的职业人才教育基地。积极发展高等教育，建成1-2所大学。统筹发展民办教育、特殊教育、民族教育等各类教育。

加强卫生计生服务。实施“健康贵港”计划，加快推进中心城区医院、乡镇卫生院、社区卫生服务中心和村卫生室建设，努力构建城乡15分钟健康服务圈。建立重大疾病保障机制，加大医疗救助力度，巩固新农合参保率。建立覆盖城乡的基本医疗卫生制度和现代医院管理制度，实现城乡居民人人享有基本医疗服务。扶持中医药和民族医药事业发展，鼓励社会办医。全面实施两孩政策，加强生育公共服务，促进人口长期均衡发展。

促进文化繁荣。加强城市文化建设，积极创建全国文明城市。打造“荷文化”品牌，继续举办贵港荷花展，争取承办全国荷花展。重视文化遗产和非物质文化遗产保护开发，推进历史文化名城建设。深入开展群众性文化活动，推进文化下乡。加强公共文化基础设施建设，建成市文化艺术中心，新建或扩建县市区图书馆、文化馆、博物馆和档案馆。发展广播电视事业，实现广播电视户户通。加强精神文明建设，引导市民自觉践行社会主义核心价值观，大力弘扬“和为贵、诚为本、干为先”的贵港精神，努力在全社会形成共同的理想信念和良好风尚。

扩大就业创业。实施更加积极的就业政策，推进大众创业、万众创新。扶持就业容量大的小微企业，创造更多就业岗位。加强就业创业服务和职业培训，帮助农村青年、农村妇女、返乡农民工创业，统筹做好高校毕业生、城乡新增劳动力、就业困难人员等群体的就业工作。五年内城镇新增就业7万人、农村劳动力转移就业17.5万人，城镇登记失业率控制在4.3%以内，动态消除城镇“零就业”家庭。

提升社会保障。实施全民参保计划，扩大社会保险覆盖范围，到2020年，城乡居民基本养老保险参保率90%以上，各项社会保险总参保人数750万人次以上。落实最低工资保障制度，健全欠薪整治机制，构建和谐的劳动关系。推进养老服务业综合改革试验区建设，建成一批规模较大、设施完善、管理规范的养老服务机构。适时提高退休人员基本养老金和城乡居民基础养老金标准。加大社会救助力度，发展社会福利和慈善事业。加快保障性住房建设，建成保障性住房和棚户区改造住房2.5万套（户）、公共租赁住房8900套，完成农村危房改造4万户以上。实施水库移民基础设施项目972个，改善库区移民生产生活条件。

深化社会治理。加强信访工作，畅通信访渠道，健全矛盾纠纷调解机制，及时解决矛盾纠纷。落实安全生产“党政同责、一岗双责”，强化企业主体责任，抓好重点领域、重点行业事故隐患排查整治和风险防控，坚决遏制较大以上事故发生。加强应急管理工作，提高防灾减灾和应对突发事件能力。开展“全区食品安全城市”创建试点工作，加强食品药品监管，确保舌尖上的安全。推进社会信用体系建设，营造诚实守信、公平公正的社会环境。深入开展“平安贵港”建设，积极构建社会治安防控体系，依法惩治违法犯罪活动，提升人民群众的安全感和满意度。

统筹推进其他社会事业。广泛开展全民健身运动，加强群众健身活动场地和设施建设。从2016年起，每四年举行一届市体育运动会。深入开展民族团结进步创建活动，依法管理宗教事务，引导宗教与社会主义社会相适应。认真做好双拥、优抚安置工作，强化国防教育，加强国防动员和国防后备力量建设。加强统计工作，强化公积金管理服务和价格监管，推进气象防灾减灾能力建设，重视对台和侨务工作，支持工会、共青团、妇联和工商联等群团组织参与社会治理，关爱妇女、儿童、老人和残疾人，统筹推进机关后勤、绩效、档案、地震、地方志等各项工作，密切与中区直部门合作，做好海关、海事、检验检疫、边检、武警、消防、农垦、邮政、通信、水文、石油化工、烟草、盐业、船舶检验、地质勘探、无线电监测等工作。

三、努力建设人民满意政府

各位代表！新的形势、新的任务和新的目标，对政府工作提出更高要求。我们将进一步加强自身建设，不忘初心，继续前进，更加勤勉尽责、务实担当，以更大的决心、更强的力度、更实的举措，全力推动贵港跨越发展。

（一）执政为民，打造服务政府

加强行政审批标准化建设，制定审批事项工作细则，规范审批行为。全面推进“六证合一”商事制度改革，促进登记注册便利化。健全审批帮办机制，推行项目审批“一个窗口受理、一站式办理、一条龙服务”。加强电子政务平台建设，推行网上预约、申请、查询等政务服务。加快建设市民中心，提高服务群众效率。扩充完善市长热线电话及微信、市政微信、政务微信、网络问政平台，确保政民互动渠道畅通。全面推行审批职能部门“说不”报告制度。推行政务公开政府信息公开，提高政府公信力和执行力。

（二）干事创业，打造责任政府

政府工作人员要恪尽职守、主动作为，坚持实字当头、干字为先，直面问题、说到做到，按照目标明确、措施明确、阶段性目标明确、责任人明确“四个明确”要求部署工作、推进工作、督查工作和进行奖惩；坚持以问题为导向，扭住问题不放，及时主动发现问题、及时主动解决问题、及时主动协调问题、及时主动报告问题，把各项工作落到实处。健全督查问责机制，坚决整肃庸政懒政怠政。健全激励机制和容错纠错机制，给改革创新者撑腰鼓劲。

（三）依法行政，打造法治政府

深入推进法治贵港建设，加强立法工作，加快制定城乡建设与管理、环境保护和历史文化保护等规章。自觉运用法治思维和法治方式推动工作，全面推行权力清单制度，做到法定职责必须为，法无授权不可为。积极推行政府法律顾问制度。自觉接受市人大及其常委会的法律监督、工作监督和市政协的民主监督，以及社会和舆论监督，让权力在阳光下运行。

（四）从严要求，打造廉洁政府

认真落实全面从严治党的要求，加强政府系统党风廉政建设，落实“一岗双责”责任制，教育和引导各级干部坚定理想信念，牢固树立政治意识、大局意识、核心意识、看齐意识。完善惩治和预防腐败机制，加强行政监察和审计监督，对公共资金、公共资源、国有资产严加监管。坚持勤俭办事，反对铺张浪费，把更多的资源和资金用在促进发展、改善民生上。始终保持反腐高压态势，坚持以“零容忍”态度惩治腐败，加强对扶贫等民生领域监督执纪问责，营造风清气正的良好氛围。

各位代表！市第五次党代会为我们描绘了全面建成小康社会的美好前景，明确了打造实力贵港、活力贵港、魅力贵港、给力贵港的奋斗目标。我们要在市委的坚强领导下，在市人大及其常委会、市政协的监督支持下，解放思想，开拓创新，抢抓机遇，奋力拼搏，一张蓝图绘到底，咬定青山不放松，不达目的不罢休，全力决胜小康、再创辉煌，推进港产城互动融合发展，为加快建成西江流域核心港口和新兴工业城市而努力奋斗！
